# Unique skull network complexity of *Tyrannosaurus rex* among land vertebrates

**DOI:** 10.1038/s41598-018-37976-8

**Published:** 2019-02-06

**Authors:** Ingmar Werneburg, Borja Esteve-Altava, Joana Bruno, Marta Torres Ladeira, Rui Diogo

**Affiliations:** 1 Senckenberg Center for Human Evolution and Palaeoenvironment (HEP) at Eberhard Karls Universität, Sigwartstraße 10, 72076 Tübingen, Germany; 2 Fachbereich Geowissenschaften der Eberhard-Karls-Universität Tübingen, Hölderlinstraße 12, 72074 Tübingen, Germany; 3 Museum für Naturkunde, Leibniz-Institut für Evolutions- & Biodiversitätsforschung an der Humboldt-Universität zu Berlin, Invalidenstraße 43, 10115 Berlin, Germany; 4 Structure and Motion Laboratory, Department of Comparative Biomedical Sciences, The Royal Veterinary College, London, United Kingdom; 5 Departament de Prehistòria, Universitat Autònoma de Barcelona, Barcelona, Spain; 6 Paläontologisches Institut und Museum der Universität Zürich, Zürich, Switzerland; 7 Department of Anatomy, Howard University College of Medicine, Washington DC, USA; 8 Department of Experimental and Health Sciences, Pompeu Fabra University, Barcelona, Spain

## Abstract

Like other diapsids, *Tyrannosaurus rex* has two openings in the temporal skull region. In addition, like in other dinosaurs, its snout and lower jaw show large cranial fenestrae. In *T*. *rex*, they are thought to decrease skull weight, because, unlike most other amniotes, the skull proportion is immense compared to the body. Understanding morphofunctional complexity of this impressive skull architecture requires a broad scale phylogenetic comparison with skull types different to that of dinosaurs with fundamentally diverging cranial regionalization. Extant fully terrestrial vertebrates (amniotes) provide the best opportunities in that regard, as their skull performance is known from life. We apply for the first time anatomical network analysis to study skull bone integration and modular constructions in tyrannosaur and compare it with five representatives of the major amniote groups in order to get an understanding of the general patterns of amniote skull modularity. Our results reveal that the tyrannosaur has the most modular skull organization among the amniotes included in our study, with an unexpected separation of the snout in upper and lower sub-modules and the presence of a lower adductor chamber module. Independent pathways of bone reduction in opossum and chicken resulted in different degrees of cranial complexity with chicken having a typical sauropsidian pattern. The akinetic skull of opossum, alligator, and leatherback turtle evolved in independent ways mirrored in different patterns of skull modularity. Kinetic forms also show great diversity in modularity. The complex tyrannosaur skull modularity likely represents a refined mosaic of phylogenetic and ecological factors with food processing being probably most important for shaping its skull architecture. Mode of food processing primarily shaped skull integration among amniotes, however, phylogenetic patterns of skull integration are low in our sampling. Our general conclusions on amniote skull integrity are obviously preliminary and should be tested in subsequent studies. As such, this study provides a framework for future research focusing on the evolution of modularity on lower taxonomic levels.

## Introduction

The hypercarnivore *Tyrannosaurus rex* (Theropoda) is an icon of paleontology and evolution. Its unique anatomy, including an immense skull and small forelimbs, has inspired a number of morphofunctional experiments^1,2^. Its skull length is about a sixth of the total body length, and cranial performance analyses resulted in reconstructions of a very powerful bite force^3–7^. In addition to the ancestral temporal fenestrae, large cranial openings in the snout and the lower jaw are thought to decrease skull weight and to better distribute strain when processing food, but also other morphofunctional reasons have been discussed such as specific muscle insertions, shock absorption, pneumaticity, and internal skull mobility^6,8,9^. As a result, the skull shows a complex architecture made of several bone bars, typical also for other theropod dinosaurs. Theropod skull diversity^10,11^, like that of dinosaurs as a whole^8^, is very high. A general understanding on the relationship between modular and functional patterns of amniote skulls is needed to understand the morphofunctional diversity of those groups.

Among fully terrestrial vertebrates (amniotes), a great diversity of skull types exists^12^. For example, the temporal skull region behind the eye can be closed, can have one or, like in tyrannosaurs, two openings, or can display marginal reductions between bones. In addition, the snout, braincase, and palatal region experienced great modifications through evolution related to behavioral (e.g., neurobiological) and ecological (e.g., feeding, fossorial) adaptations^13^.

Here we present the first study of the cranial complexity and modularity of *T*. *rex* in comparison with a sample representing all the major clades of extant amniotes (lepidosaurs, turtles, crocodylians, birds, and mammals) using Anatomical Network Analysis (AnNA)^14,15^. This methodology allows us to detect deeper anatomical aspects that cannot be examined otherwise and can provide crucial information about potential functional entities. AnNA complements the more traditional approach to morphological integration and modularity using morphometrics, which focuses on co-variations in size and shape^16^. Specifically, AnNA uses algorithms from network theory to quantify and compare the anatomy of organisms^14,15^. This approach starts with the modeling of anatomical structures as networks, in which nodes represent anatomical parts (e.g., bones) and links represent their physical connections (e.g., articulations).

**Figure 1:**
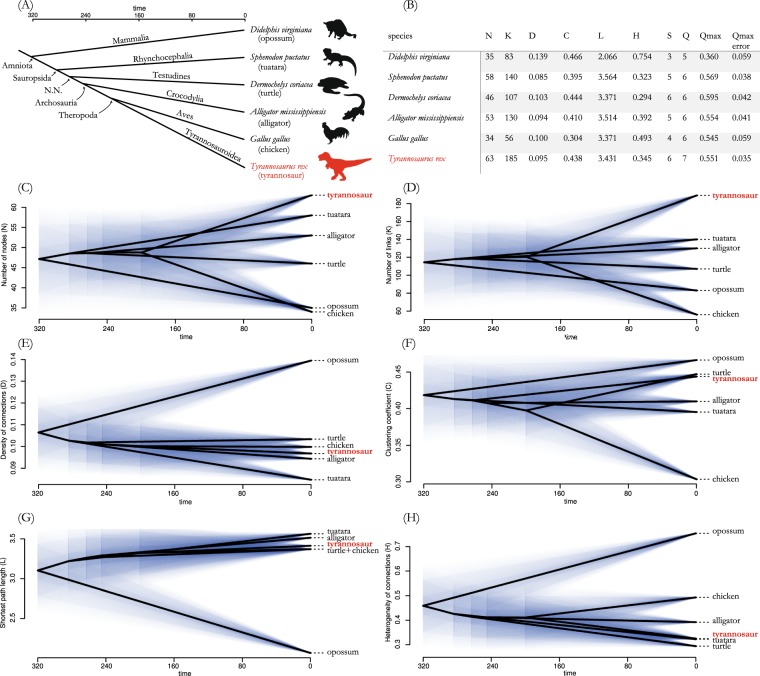
Phylogenetic arrangement of taxa analyzed in this study. Phylogenetic framework and divergence times correspond to Shedlock and Edwards^75^ for extant taxa and Benton^76^ for *Tyrannosaurus rex* (**A**). Values of network parameters and results of the modularity analysis for every skull network in the sample (**B**). Phylograms of network-parameters (**C**–**H**). Abbreviations within the subfigures: N, nodes, number of skeletal elements; L, links, number of contacts among skeletal elements (i.e., physical joints); (**D**) density of connections, actual number of links divided by the maximum number possible; (**C**) mean clustering coefficient, average of the ratio of a node’s neighbors that connect among them; L, mean shortest path length, average number of links required to travel between two nodes; H, heterogeneity of connections, standard deviation divided mean of the number of connections of every node; S, number of S-modules, modules identified using statistical significance; Q, number of Q-modules, modules identified using optimization function Q; Q_max_, maximum value of Q calculated for the best partition of the dendrogram; Q_max_ error, expected error of Q_max_. Time in million years.

We hypothesized that life performance of the tyrannosaur skull can be deduced from patterns of bone integration in extant amniote skulls, for which functional morphology is better understood than in any fossil. To better understand the skull of *T*. *rex*, here we provide a function-related discussion of modularity in an evolutionary context^17^. As representatives of the major extant amniote clades, we studied skull networks of the Virginian opossum, tuatara, leatherback turtle, alligator, and the chicken, which in our sampling represents the closest relative of tyrannosaurs (Fig. 1A).

## Results

### Network parameters describing skull anatomy

*Tyrannosaurus rex* and chicken have the skulls and lower jaws with the highest and lowest number of bones of the sample (N = 63 and 34, respectively) (Fig. 1B). The number of bones in the chicken is close to that of the opossum (N = 35), whereas tyrannosaur is closer to the other diapsids: tuatara (N = 58) and alligator (N = 53) (Fig. 1C). The leatherback turtle takes intermediate values (N = 46). The same pattern observed for nodes is visible for the number of links (connections between bones: K) (Fig. 1D).

When considering the density of connections (D), tyrannosaur (D = 0.095) falls close to chicken and other sauropsids (i.e., reptiles), as is expected by its phylogenetic position (Fig. 1A,E). The parameter D has been interpreted in past works as a proxy of morphological complexity, with a higher value of D meaning a greater complexity of the skull^18^. In this regard, the chicken skull is as complex as that of other sauropsids, while the opossum (D = 0.139) has the skull with the greatest morphological complexity of all.

The chicken shows a relatively low mean clustering coefficient (C = 0.304) that sets it apart from the other taxa of the sample, with about 10% less C than others. Tyrannosaur (C = 0.438), however, falls close to the leatherback turtle (C = 0.444) (Fig. 1B,F). C has been interpreted in the past as a proxy of anatomical integration, with higher values of C meaning greater integration^18^. In this context, integration refers to the degree of inter-connectivity among the parts of the skull; having a greater integration is related to stronger constraints or co-dependences, such as those affecting co-evolution or co-variation of skull regions^15^. This means that chickens have evolved a less integrated skull anatomy.

All taxa except the opossum show a similarly large mean shortest path length (L), although opossum has a relatively small value (L = 2.066) (Fig. 1G). The shortest path length is a network parameter or topological variable. It measures the minimum amount of connections separating two nodes (e.g., two connected nodes have an L = 1; if they are not directly connected, L will be >1; thus, the higher their L the more separated they are). L has been interpreted in the past as proxy of integration, with smaller values of L meaning greater integration^18^. In this context, the chicken skull (L = 3.371) is less integrated than those of reptiles, while the opossum skull is the least integrated of all. The tyrannosaur (L = 3.431) falls together with all other sauropsids, clearly deviating from the opossum. As stated above, overall skull integration at a topology has been suggested to affect other aspects of phenotypic organization and variation^19^.

Regarding heterogeneity of connections, the chicken (H = 0.493) shows intermediate values between the other sauropsids and the opossum (Fig. 1B,H). H has been interpreted as a proxy of anisomerism (i.e., irregularity, differentiation, or specialization) with higher H meaning greater anisomerism^18^. In this context, chicken skull bones have a degree of differentiation (in terms of connectivity) between other sauropsids (low) and the opossum (high), with tyrannosaur (H = 0.345) falling together with the chicken and the other reptiles, with values similar to that of tuatara (H = 0.323), which would suggest a phylogenetic linkage.

### Modularity

**Figure 2:**
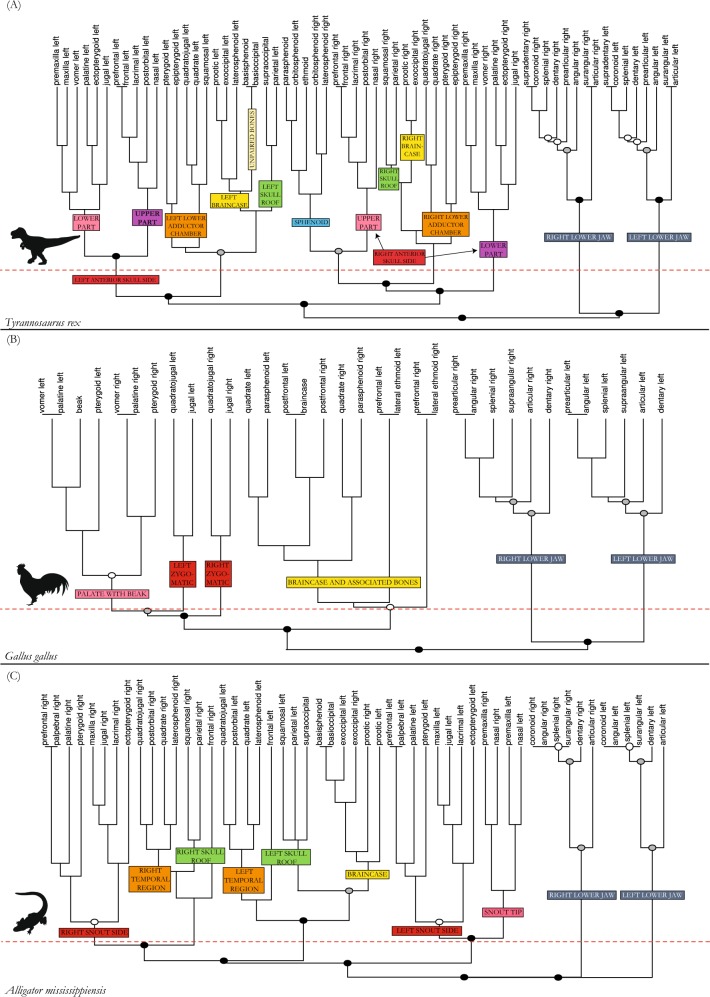
Dendrograms showing the hierarchical clustering of nodes for each anatomical network in tyrannosaur (**A**), chicken (**B**), and alligator (**C**). Anatomical regions (modules/submodules) are labeled on the branches. The horizontal red dashed line marks the best partition into Q-modules. Cluster with a circle are all S-modules; filled circles in clusters mark the statistical significance of this potential module (white, p-value < 0.05; grey, p-value < 0.01; black, p-value < 0.001).

**Figure 3:**
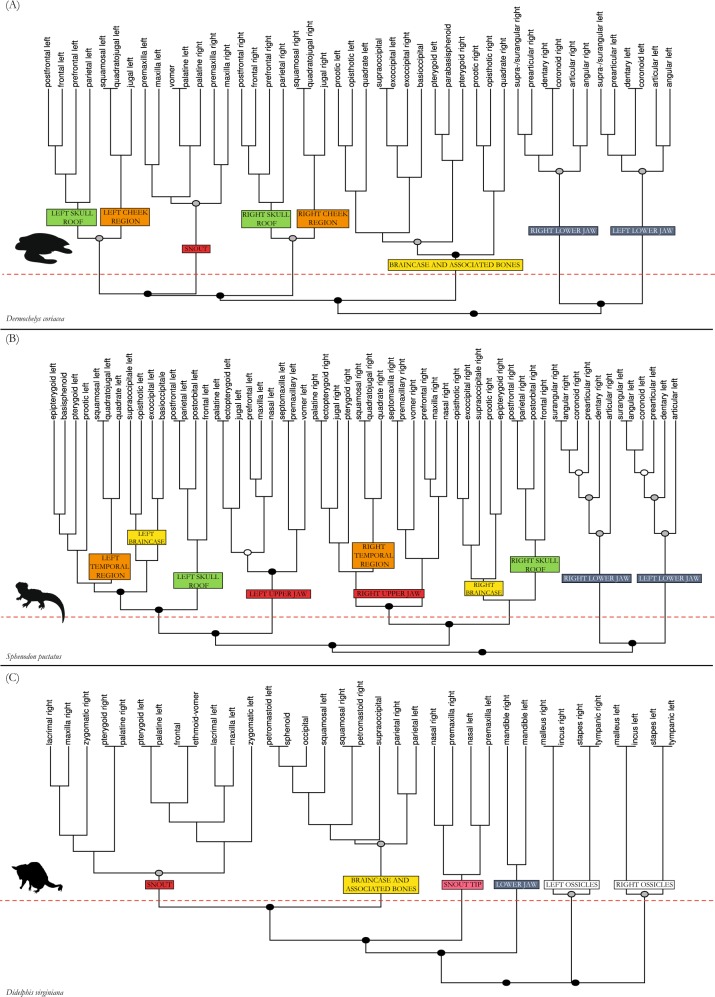
Dendrograms showing the hierarchical clustering of nodes for each anatomical network in leatherback turtle (**A**), tuatara (**B**), and opossum (**C**). Anatomical regions (modules/submodules) are labeled on the branches. The horizontal red dashed line marks the best partition into Q-modules. Cluster with a circle are all S-modules; filled circles in clusters mark the statistical significance of this potential module (white, p-value < 0.05; grey, p-value < 0.01; black, p-value < 0.001).

**Figure 4:**
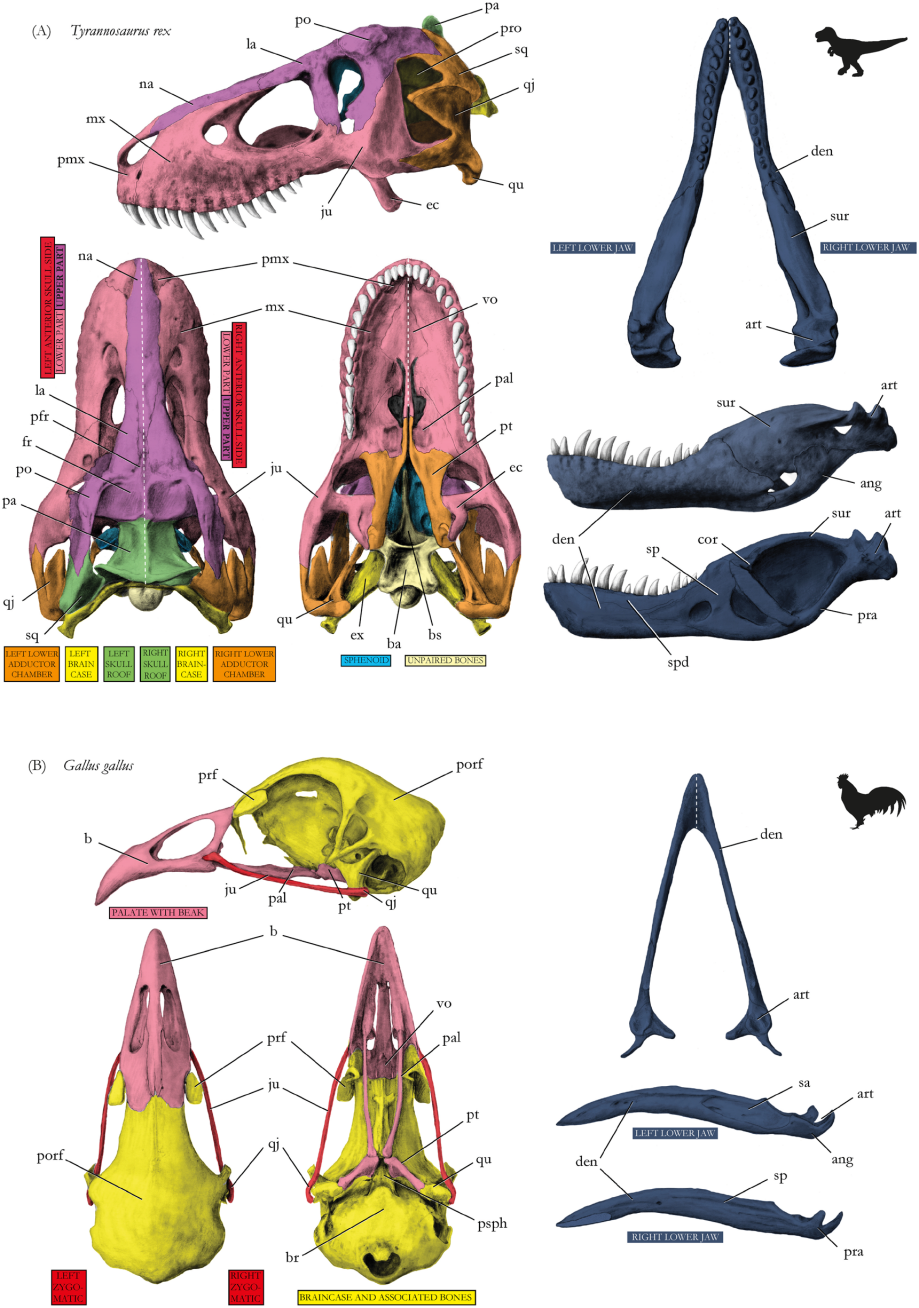
Drawings of skulls with modules shown in different colors corresponding to Fig. 2A and B, respectively. (**A**) Tyrannosaur *Tyrannosaurus rex*, (**B**) chicken *Gallus gallus*. Abbreviations for Figs 4–6: ang, angular; art, articular; b, beak; ba, basioccipital; br, braincase; bs, basisphenoid; cor, coronoid; den, dentary; ec, ectopterygoid; epi, epipterygoid; et, ethmoid; fr, frontal; ju, jugal; la, lacrimal; mx, maxilla; na, nasal; oc, occipital; op, opisthotic; pa, parietal; pb, palpebral; pal, palatine; pbs, parabasisphenoid; pe, petromastoid; pfr, prefrontal; pmx, premaxilla, po, postorbital; pofr, postfrontal; pra, prearticular; pro, prootic; psph, parasphenoid; pt, pterygoid; qj, quadratojugal; qu, quadrate; sa, supraangular; smx, septomaxilla; so, supraoccipital; sp, splenial; sph, sphenoid; sur, surangular; vo, vomer; zy, zygomatic. For drawings without coloration, see Supplementary Figures.

The tyrannosaur shows a more modular skull and has, in terms of network theory, a less consistent pattern (Q-modules = 7, Q_max_ = 0.551, Q_max.error_ = 0.035) than any other amniote studied here (Q-modules = 5 or 6; Figs 1–3). An internal separation of the left anterior skull side exists (red module in figures, p = 0.001; Figs 2A and 4A) consisting of upper jaw and anterior palatal bones in its lower part (pink module) and anterior skull roof and circumorbital bones in its upper part (purple module, p = 0.001). Posterior skull roof bones (green module) and braincase bones (yellow module) form modular patterns on each side. The sphenoids and the ethmoid form a close association (blue module). A close relation of bones situated in the lower part of the adductor chamber (orange module), including quadrate, palatoquadrate, pterygoid, and epipterygoid, exists on each side. In contrast to all other amniotes studied, the detailed composition of bones in the modules of the tyrannosaur skull is very inconsistent on each side and the association of modules differs as well. This means that modules are largely independent from each other and internal integrity is not as consistent/stable in a topological sense.

In the chicken, the palate and beak form one single module (pink in Fig. 2B), the left and the right zygomatic arch form distinct modules (red), and all other bones, surrounding and including the braincase, form the other module (yellow) (Figs 2B and 4B).

**Figure 5:**
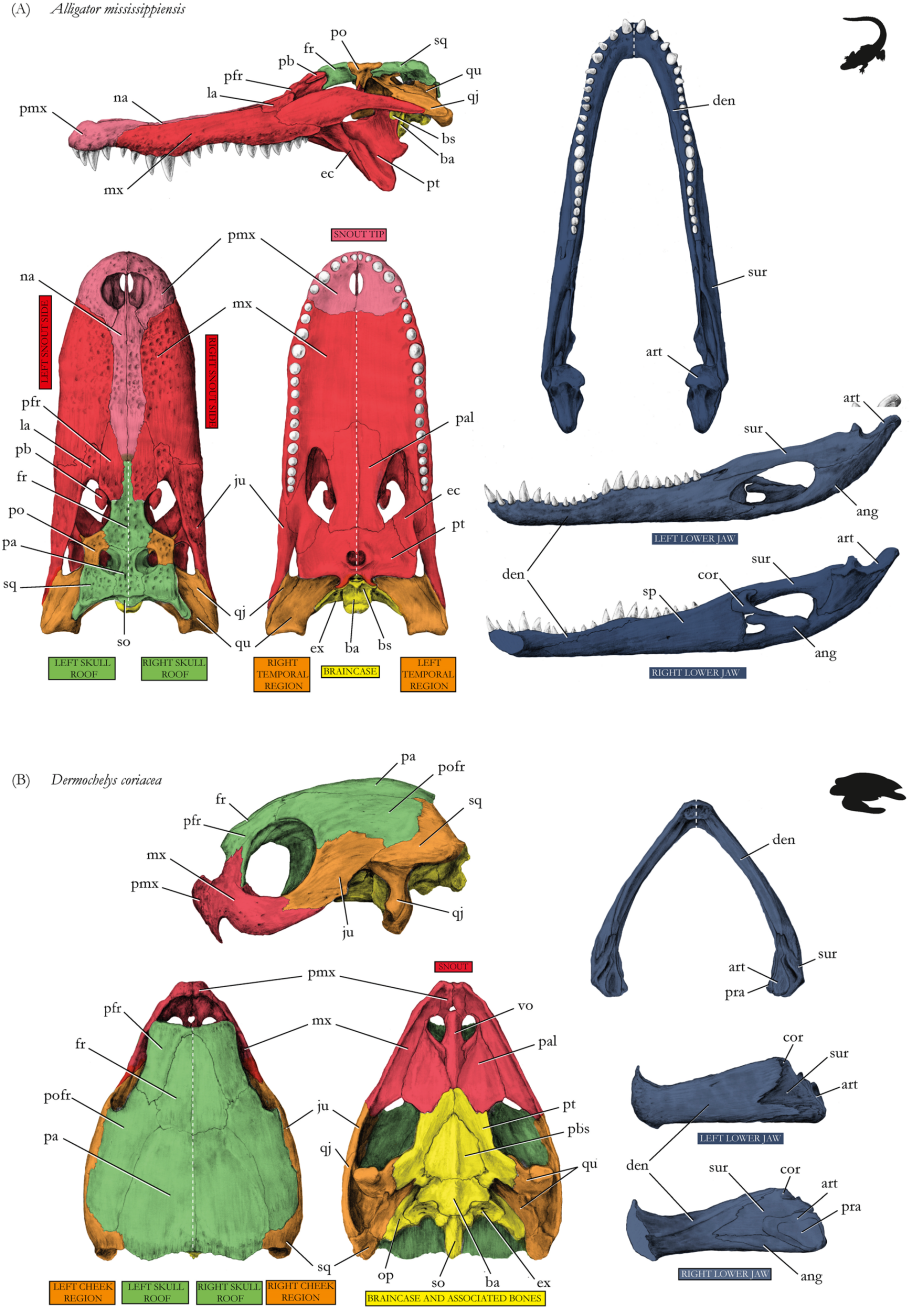
Drawings of skulls with modules shown in different colors corresponding to Figs 2C and 3A, respectively. (**A**) Alligator *Alligator mississipiensis*, (**B**) leatherback turtle *Dermochelys coriacea*. For abbreviations, see caption of Fig. 4. For drawings without coloration, see Supplementary Figures.

**Figure 6:**
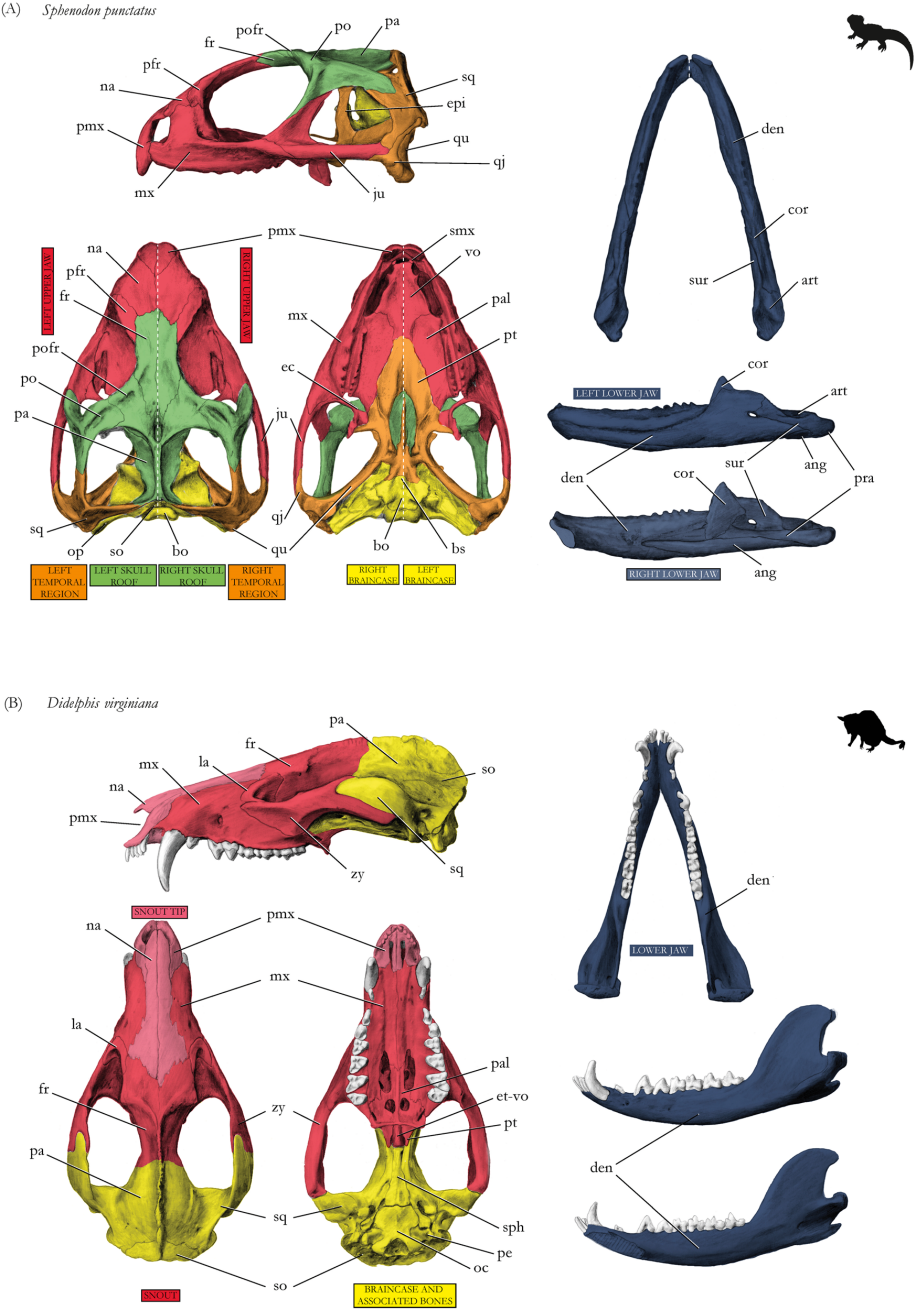
Drawings of skulls with modules shown in different colors corresponding to Fig. 3B,C, respectively. (**A**) Tuatara *Sphenodon punctatus*, (**B**) opossum *Didelphis virginiana*. For abbreviations, see caption of Fig. 4. For drawings without coloration, see Supplementary Figures.

In alligator, the left and right snout parts are split into two separate modules (red in figures, p = 0.05). Anteriorly, the snout tip forms a separate module (light red) (Figs 2C and 5A), like in the opossum (Figs 3C and 6B), being limited posteriorly by canine-like large teeth from the lower jaw, and bearing the nose opening dorsally (Fig. 5A). Similar to tuatara (Figs 3B and 6A), the temporal bones of alligator form a module (orange) within a higher integration of other skull elements on each side. The pterygoid is part of the module that comprises the large snout related bones (red). The small braincase (yellow) is loosely integrated to the temporal and the skull roof region.

The leatherback turtle shows a separation in a bilateral snout module (red in figures), a left and a right module (p = 0.01) combining the skull roof (green) and the cheek bones (orange), and a module composed of braincase and associated elements (yellow) (Figs 3A and 5B).

The tutatara has a clear modular pattern distinguished into left and right sides of the skull (Figs 3B and 6A). Temporal bones (orange in figures), skull roof bones (green), and braincase bones (yellow) are modular entities in tuatara, nested within each skull side, although they do not show a clearly positioned integration to each other.

The opossum shows one module made of snout bones from both body sides (red in figures) (p = 0.01), including palatal and upper jaw elements (Figs 3C and 6B). The posterior part of the skull with the braincase and associated bones, such as the squamosals and parietals, form the second module (yellow). The tip of the snout, as a third module (light red), is made of the nasals and premaxillas. The ear ossicles form completely separated modules on each side (white, p = 0.01), because they are not connected to other bones.

In addition to the above-mentioned modules of the skull, each sauropsid has two separated lower jaw modules with species-specific integration of the particular bones (grey in figures). Except for the beak-bearing chicken, the articular, which articulates with the quadrate, is less integrated to the other lower jaw elements. Importantly, in the opossum both mandibles (dentaries) form a single module (grey), providing a further example of a phylogenetic pattern (i.e., contralateral bone fusion in mammals) revealed by AnNA. The tyrannosaur does not show a particularly different modular system although a large fenestra is present on the mesial side of its lower jaw.

## Discussion

The tyrannosaur has the most complex and least consistent modular pattern among the sample representatives of each major extant amniote clade. In terms of its topology, the tyranosaur skull is clearly peculiar, displaying a high number of modular subdivisions. Single bones are not necessarily fixed to a particular module. Moreover, modules are largely independent from each other on each body side. These important findings suggest a highly flexible skull, in terms of functional morphology. We detected diverse modular patterns for all amniotes studied here. These patterns are highly specific for each taxon and are likely related to specific functional demands, because despite some clear cases of phylogenetic patterns that were revealed by AnNA, only little phylogenetic correlation was detected (Fig. 1B). The comparative discussion of skull modularity among amniotes, highlighting common and diverging patterns, permits us to provide here a refined morphofunctional characterization of the tyrannosaur, a taxon deeply nested within amniote phylogeny (Fig. 1A).

### Bone reduction

The number of bones in the tyrannosaur is similar to that of all other sauropsids except for the chicken. Through fusion processes during ontogeny, this relatively close relative of the tyrannosaur shows great reduction of ossification centers^20^ and, convergent to opossum, accordingly shows great bone reduction (low N- and K-values). The complexity of the chicken skull (estimated by D) is, however, similar to that of other sauropsids illustrating that bone connections were established differently when compared to opossum. This observation is further supported by the low integration (as estimated by C and L) in chicken, showing that bone contacts arranged in a different, less compact way when compared to those of the opossum. In the latter taxon, inter-connections among close bones produced a very compact skull (high D and C, low L) and although heterogeneity (H-value) is higher in the chicken when compared to other sauropsids, it is still far from that achieved in opossum. The higher compactness in opossum, however, does not necessarily imply less modularity – which is similar in chicken and opossum (i.e., number of Q-modules, Fig. 1B) – meaning that individual bone size and anatomical differentiation are, of course, also important to establish modular patterns in the skull.

### Kinetic and akinetic forms

Kinesis of the skull not only refers to mobile joints between bones, but cranial kinesis can also be developed in skulls with immobile sutures by having relatively thin and elastic bones in gracile crania that eventually enable the same range of motion^21^. Cranial kinesis of tuatara is highly disputed. Although a single cineradiography experiment failed to demonstrate (joint based) cranial kinesis^22^, anatomical conditions such as the presence of constrictor dorsalis musculature, indicate that at least some degrees of skull kinesis are actually present in this species^23–25^. Kinesis appears to be largely reduced in adult tuatara by strong sutures between bones, although the gracile architecture of the skull is generally compatible with elasticity-related cranial kinesis, particularly in juveniles, which have a different feeding behavior compared to adults^25^. Given the lightweight design of the tuatara skull^26^, we define it as kinetic herein. Also dinosaurs do not have well-developed intracranial joints when compared to many lizards or birds; however, little is known about actual intracranial mobility in their comparably gracile skulls^23^.

In this regard, we found a clear distinction between species with a suspected skull kinesis (i.e., tyrannosaur, chicken, tuatara) and species with fully akinetic skulls (i.e., alligator, leatherback turtle, opossum). Akinetic species, have to withstand extremely strong biting forces^27–29^. As a result, they have fewer bones (N ≤ 53) than tyrannosaur and tuatara and relatively high network density, meaning they have to build broader sutures (e.g., in contrast to tuatara) to each other to keep integrity. Suspected kinetic species have more bones (N ≥ 58) and, in average, a lower network density (Fig. 1), meaning that, overall, smaller sutures might enable high mobility in the articulations and/or bones are more flexible/elastic than in akinetic species. Among kinetic species, however, the tyrannosaur has the greatest density, a bit larger than the akinetic alligator, which appears to relate to its supposedly hypercarnivor behavior and powerful food processing. The chicken skull is an exception in this regard, because it has fewer bones (N = 34) but, as mentioned above, its network density is relatively high. This is because its braincase bones are highly fused to support the brain^30,31^; the remainder of the skull, including palate, break, and skull roof, consists of very fragile and highly kinetic elements^32^. Overall skull network construction is highly diverse, and has little affinity to phylogenetic relationship as noted above. Even if the turtle and archosaurs (tyrannosaur, chicken, alligator) are relatively closely related^33^, the physical joints differ in number and position. The akinetic skulls obviously evolved independent from each other in these lineages.

### Individual skull modularity

Patterns of modularity show a clear correspondence to skull architecture in each species. We first discuss the modularity of opossum, as the mammalian outgroup, and then continue along the tree (Fig. 1A) with tuatara, leatherback turtle, alligator, and finally chicken as the taxon more closely related to the tyrannosaur. By doing this comparison, we hope to contribute to a better understanding of the evolutionary history of tyrannosaur skull complexity and modularity.

The integration of left and right body side elements in individual bilateral modules nicely illustrates the compact nature of the akinetic mammal skull, as seen in the example of opossum (Figs 3C and 6B). In mammals such as the opossum food processing occurs in the middle of the snout module. The snout includes the zygomatic bone (Fig. 6B), from which the chewing muscle, the masseter, mainly originates. The main bite forces appear in the posterior part of the skull, from which the jaw closing muscle, the temporalis, originates^34^. Associated to that, the sphenoid, which braces the lateral braincase^35^, is part of the posterior skull module (Figs 3C and 6B). The separate module at the snout tip corresponds to the macrosmathic nature of most mammalian skulls. The canines, which have to resist much biting force^36^, are situated posterior to this module.

Tuatara shows a very independent nature of its modules in the global context of the gracile skull, the architecture of which is made of several bony bars, loosely embracing the modules. The skull of tuatara is principally constructed as kinetic (see above) and the certain independency of modules (Figs 3C and 6B) might enable a better flexibility when processing food^37^. For that, highly differentiated jaw adductor musculature of lepidosaurs allows for handling agile prey^23,38,39^. The epipterygoid (Fig. 6B), which supposedly moves the palatal shelve in lizards, is a part of the braincase module in tuatara, which also highlights the diverse labor division in the adductor chamber.

The snout of turtles is very broad and left and right sides are strongly fused, likely as an adaptation of the akinetic skull. Early in turtle evolution, the skull was still kinetic as indicated by a pterygoid-braincase articulation. In extant turtles, without such articulation, the pterygoid (Figs 2C and 5B) is an integrated part of the braincase module^40–42^. In addition, the braincase is broadly fused with the quadrate in crown turtles making cranial mobility impossible^41,43,44^. In crown turtles, development of the akinetic skull is completed by the formation of a secondary lateral braincase wall, mainly made of a descending process of the parietal^45^. As such, the skull roof - present as a submodule in the leatherback turtle - also covers the brain laterally. The cheek submodule is composed of jugal, quadratojugal, and squamosal (Figs 3A and 5B) bones to which the superficial external adductor musculature attaches^46–49^.

The alligator also has an akinetic skull. However, it has evolved a different modular pattern of skull bones compared to the opossum and leatherback turtle, and a unique mode of skull fixation took place^50^. Like in opossum, the snout tip is not as much involved in bite force distribution as the rest of the snout^19^, making it a separate module (Figs 2C and 5A). Apparently, the double-fenestrated temporal region in the alligator resulted in a separate temporal module such as in tuatara. In both species, the external jaw musculature attaches to the temporal region in a complex manner^51^. In alligator, however, most biting force is transmitted via pterygoid-related internal jaw musculature making the pterygoid a part of the snout module^52,53^.

The specific avian adaptation of having both large eyes and beaks is clearly mirrored in the detected modularity (Figs 2B and 4B). The posterior part of the skull forms a dense capsule of bones^31^. The kinetics are highly derived when compared to the ancestral diapsid condition (with two temporal openings), which is still partly preserved in tuatara^37^, and the beak is moveable against the skull roof (prokinesis) thanks to the lever of the zygomatic arch^32^.

### Tyrannosaur skull performance

The highly complex modularity detected for the tyrannosaur (Figs 2A and 4A) indicates a very flexible skull regarding bite force distribution. Although loosely associated to others, the best-resolved modular associations are established by the lateral sides of the skull. The comparison to other amniotes and biological considerations result in the following assumptions.

1. Like all supposedly or clear kinetic forms studied herein (tuatara, chicken), a high degree of cranial mobility is possible in the tyrannosaur when compared to clear akinetic forms. This is consistent with the flexible sutures^11^ and the great number of links in the network of its cranial bones. This flexibility should be taken into account when reconstructing cranial performance^54^. Like in alligator, a large snout is formed; however, different to alligator, streptostylic movement (via the epipterygoid, which connects the laterosphenoid and the quadrate process of the pterygoid) might have existed in the tyrannosaur. Although quite robust^8^, the epipterygoid might have enabled some independent movements of the snout against the rest of the skull, like in tuatara, and an internal mobility between the upper and lower snout regions might have been beneficial in prey processing.
2. Internal snout mobility is supported by the formation of large fenestrations in the snout, which are mainly thought to enable pneumaticity, reduction of skull weight, and to buffer bite forces^6,9^, but also reduces the contact area between bones and as such triggers higher mobility between them. The modular separation of the snout in upper and lower parts, related to snout fenestrations, not necessarily mirrors bite force distribution. It was reconstructed to travel from the canine-like teeth upwards when puncturing prey^6,10^, a pattern also found in mammals, which do not have such a facial fenestration^55^. The tyrannosaur was hypothesized to have not only punctured but also pulled its food^6,10,54^. Associated to the presence of thecodont teeth, nesting in jaw alveoles, strain travels mainly along the upper jaw via the maxillar-jugal contact^6^ in the lower part of the snout module. Pulling food, as such, might have had a major influence in separating a lower and an upper snout submodule. In this context it is worth noting that, in contrary to the opossum, although specialized teeth are present on the premaxillary, no snout tip module was formed. Finite element analyses might be able to confirm this refined hypothesis for tyrannosaur when considering suture anatomy [*sensu*^56^].
3. Although having a typical diapsid skull (like tuatara and alligator), a separation of a temporal bone module does not exist in tyrannosaur. This indicates that bite force might have been concentrated on a different aspect of the skull, namely the lower part of the adductor chamber, to which the muscles adductor mandibulae posterior and pseudotemporalis insert^57–59^. The lower adductor chamber represents a separate module in our analysis. Voluminous jaw muscles have also been reconstructed for the upper part of the adductor chamber. However, within particular muscles, the distribution of different muscle fiber types^60,61^ and different fiber orientation^62^, which generate specific force distributions, were not yet considered in any finite element analysis. Moreover, reconstructing fiber distribution in extinct species would be extremely time consuming. As such, AnNA, as presented here, in combination with biomechanic analysis is crucial to provide a first approximation to a more reasonable reconstruction of feeding biology in tyrannosaurs - and other fossil vertebrates.

## Conclusions

In summary, the results of this study highlight how the use of AnNA can offer a new perspective on long-standing anatomical problems and help to better understand the evolvability of amniote skulls and their adaptations to different feeding behaviors. “Hidden” kinetics, not obvious due to the absence of movable joints but enabled by elastic bone and gracile skull constructions permit better estimation of skull performances. Using the extant bracketing approach and broad scale phylogenetic comparison helps understanding functional constraints and possibilities in extinct species for which no direct data on life performances are available. Our results show a relatively low correlation between the network skull integration and modularity with phylogenetic relationships, which suggest that the patterns of integration and modularity revealed in the present work are more likely related to ecological factors, such as the mode of food processing. In all species, however, a trade-off exists between phylogenetic and general functional constraints, such as lowering skull weight in tyrannosaur or brain encapsulation in birds on the one hand, and feeding adaptations on the other hand. Future studies may concentrate on modular patterns on lower taxonomic levels, for example, comparing different non-avian theropod or bird species to test the general conclusions of our work and to specify functional integrity of specific skull regions. Moreover, studies relating muscle and bone integration will help detailing the functional derivations hypothesized herein. We believe this founding study will pave the way for more comprehensive analyses of reptile evolution in particular and of anatomical evolution in general, and for the further use of AnNA to tackle morphological complexity, integration, and modularity in evolution.

## Methods

### Taxonomic sampling

We selected a sample of species from all major amniote groups for the analysis, including the opossum *Didelphis virginiana*^63^ (Mammalia), the tuatara *Sphenodon puctatus*^25,38,64^ (Lepidosauria), the leatherback turtle *Dermochelys coriacea*^65^ (Testudines), the alligator *Alligator mississippiensis*^66,67^ (Crocodylia), the chicken *Gallus gallus*^20,30^ (Aves), and the tyrannosaur *Tyrannosaurus rex*^68–70^ (Tyrannosauridae).

For *D*. *virginiana*, the sphenoid was coded as a single bone because its subparts (e.g., the alisphenoid) are not completely separated (by sutures) bones in the adult (for a recent review, see^63^]. *D*. *coriacea* is a representative of cryptodire turtles with a fully formed dermal armor above the adductor chamber. In this regard, it resembles the morphotype of stem turtles such as *Proganochelys quenstedt*^40^. However, it must be noted that skull diversity is enormous among turtles and marine turtles such as *D*. *coriacea* show a secondary formation of a full temporal armor^12^. Development and fusion of ossification centers is well traceable in *G*. *gallus*^20^. In the adult condition (coded herein), however, the braincase shows comprehensive fusion.

### Anatomical network analysis

We built unweighted, undirected network models of the skull anatomy of the six amniote taxa. Anatomical networks were coded as adjacency matrices in Excel sheets, with each contact between two nodes coded as “1” and the absence of contact between those nodes coded as “0”. In defining nodes and links, we did not include cartilaginous elements because this information is not known for fossils and/or homology of cartilaginous elements is not fully resolved in most taxa. Data were analyzed with R^71^ using the package *igraph*^72^. For every network, parameters were measured using functions implemented in the package *igraph* (see below and Supplementary information).

### Identification of network modules

We delimited the modules of the anatomical networks using a hierarchical clustering of the generalized topological overlap similarity matrix among nodes (GTOM)^14,73^. The heuristic of GTOM is that nodes that connect to the same other nodes (i.e., share neighbors) have a higher chance of belonging to the same module. Topological overlap between two nodes is defined by the number of common neighbors between them as, $T{O}_{i,j}=J({n}_{i},{n}_{j})/\mathop{\min }\limits_{k}({k}_{i},{k}_{j})$, where *J(n*_*i*_, *n*_*j*_) is the number of neighbors in common between nodes *i* and *j*. TO is 1 when the two nodes share all their neighbors, that is, they connect to exactly the same other nodes. TO is 0 when the two nodes have no neighbor in common. Using the values of TO as a distance or dissimilarity matrix, we then group nodes into clusters using an agglomerative hierarchical cluster analysis (do not confuse this with the parameter clustering coefficient). The output is a hierarchical grouping of nodes as in a dendrogram.

To identify the modules of the network we needed to cut the dendrogram at an appropriate height. To make this decision we used the optimization function modularity Q defined by Clauset *et al*.^74^, which is commonly used to assess whether the best partition identified by a community detection algorithm is better that what is expected at random. For each possible partition of the dendrogram we measured Q, the best partition is the one having the highest Q or Q_max_ (dashed red lines in Figs 2 and 3). Thus, quality of the identified partitions is given by its Q value: Q will be close to 0, if the number of links within modules is no better than that expected at random; Q will be closer to 1 if the modules identified deviate from what is expected for a random network. According to Clauset *et al*.’s^74^ observations, usual values of strongly modular networks range between 0.3 and 0.7. The expected error of Q was calculated using a jackknife procedure, where every link is an independent observation. Modules identified using the cutting of the dendrogram at Q_max_ were called Q-modules.

We also calculated the statistical significance of every cluster of the dendrogram. This was done by performing a two-sample Wilcoxon rank-sum test on the internal vs. external connections of every module. According to the general definition of a module as a group of nodes highly connected among themselves and poorly connected to nodes in other groups, we expect internal connections to be significantly higher than external connections (H_0_: K_internal_ = K_external_; H_A_: K_internal_ > K_external_). Lower p-values tell us to reject H_0_, and hence we can assume the alternative hypothesis, that the nodes of the module are more connected among themselves than to other nodes outside the module, is supported. In other words, the module identified is not expected by a random grouping of nodes. Figures 2 and 3 show the statistical significance of each cluster using colored circles (no-circle, p-value ≥ 0.5, white, p-value < 0.05; grey, p-value < 0.01; black, p-value < 0.001). Modules identified using the p-value of the two-sample Wilcoxon rank-sum test were called S-modules. For a meaningful biological interpretation, a balanced discussion between Q- and S-modules is necessary.

## Supplementary information

Online Supplement 1–4.pdf

## Data Availability

Four online supplementary files are associated to this paper.
