# Supplementary material for: Unique skull network complexity of Tyrannosaurus rex among land vertebrates
Source: Sci Rep. 2019 Feb 6;9:1520. doi: 10.1038/s41598-018-37976-8 (PMC6365547; doi:10.1038/s41598-018-37976-8)
Supplement: Supplementary file 1 — Online Supplement 1–4.pdf [file 41598_2018_37976_MOESM1_ESM.pdf]

Supplementary data from:

Unique skull network complexity of *Tyrannosaurus rex* among land vertebrates

by Ingmar Werneburg, Borja Esteve-Altava, Joana Bruno, Marta Ladeira, Rui Diogo

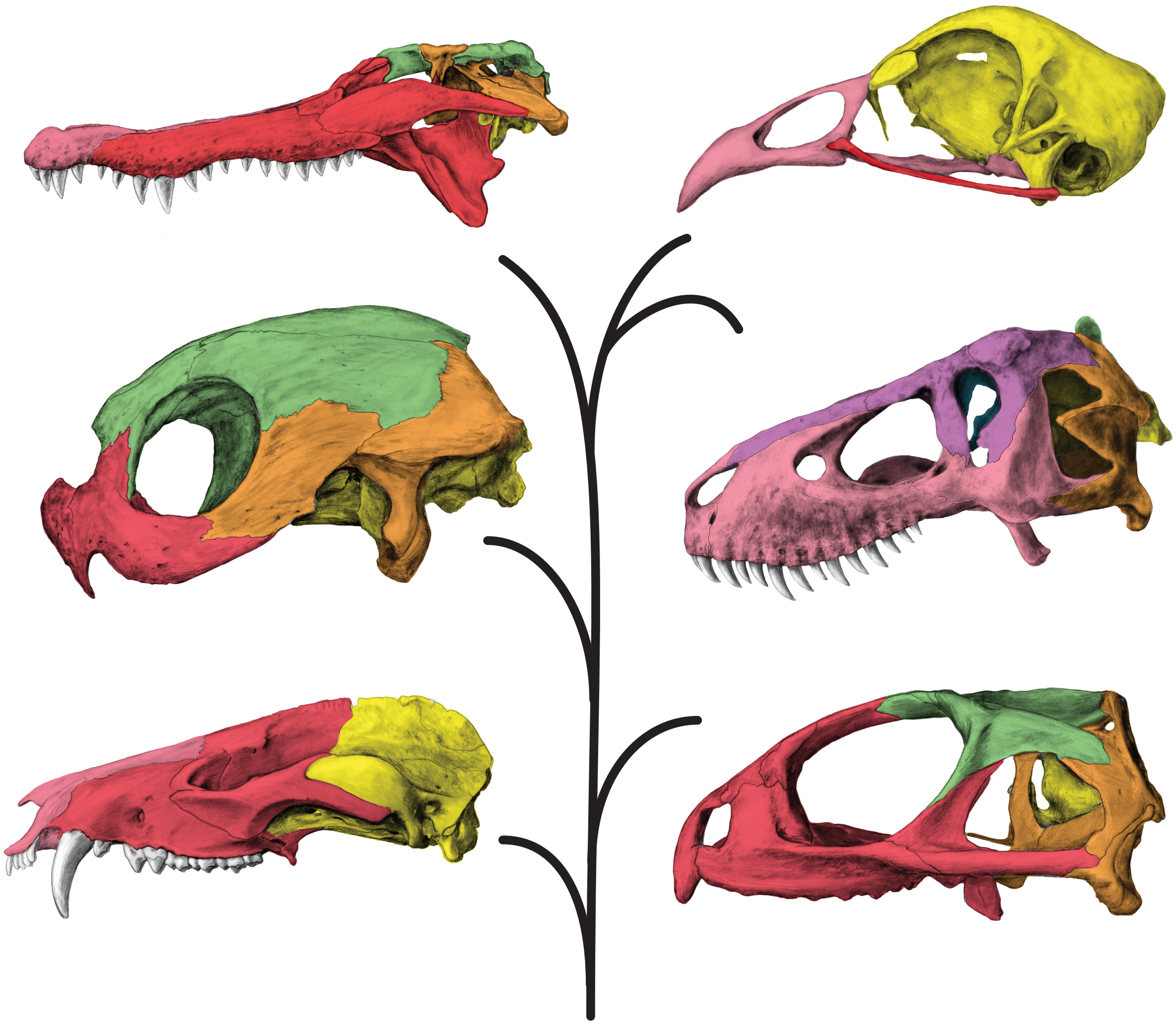

Online Supplement 1. Data Matrices

Tyrannosaurus rex	angular left	angular right	articular left	articular right	basioccipital	basisphenoid	coronoid left	coronoid right	dentary left	dentary right	ectopterygoid left	ectopterygoid right	epipterygoid left	epipterygoid right	ethmoid	exoccipital left	exoccipital right	frontal left	frontal right	jugal left	jugal right	lacrimal left	lacrimal right	laterosphenoid left	laterosphenoid right	maxilla left	maxilla right	nasal left	nasal right	orbitosphenoid left	orbitosphenoid right	palatine left	palatine right	parasphenoid	parietal left	parietal right	postorbital left	postorbital right	prearticular left	prearticular right	prefrontal left	prefrontal right	premaxilla left	premaxilla right	prootic left	prootic right	pterygoid left	pterygoid right	quadrate left	quadrate right	quadratojugal left	quadratojugal right	splenial left	splenial right	squamosal left	squamosal right	supradentary left	supradentary right	supraoccipital	surangular left	surangular right	vomer left	vomer right
angular left									1																														1														1							1			
angular right										1																														1														1							1		
articular left																																							1										1											1			
articular right																																								1										1											1		
basioccipital						1										1	1																												1	1																	
basisphenoid					1											1	1							1	1					1	1			1											1	1	1	1															
coronoid left									1																														1														1				1			1			
coronoid right										1																														1														1				1			1		
dentary left	1						1			1																																											1				1			1			
dentary right		1						1	1																																													1				1			1		
ectopterygoid left																				1						1																					1																
ectopterygoid right																					1						1																					1															
epipterygoid left																								1																					1		1		1														
epipterygoid right																									1																					1		1		1													
ethmoid																		1	1									1	1	1	1			1																													
exoccipital left					1	1																		1											1										1				1						1				1				
exoccipital right					1	1																			1											1										1				1						1			1				
frontal left															1				1			1		1				1		1				1	1		1				1																						
frontal right															1			1					1		1				1		1			1		1		1				1																					
jugal left											1											1				1						1					1														1												
jugal right												1											1				1						1					1														1											
lacrimal left																		1		1						1		1				1					1				1																						
lacrimal right																			1		1						1		1				1					1				1																					
laterosphenoid left						1							1			1		1												1				1	1		1								1																		
laterosphenoid right						1								1			1		1												1			1		1		1								1																	
maxilla left											1									1		1						1				1											1																			1	
maxilla right												1									1		1						1				1											1																			1
nasal left															1			1				1				1			1												1		1																				
nasal right															1				1				1				1	1														1		1																			
orbitosphenoid left						1									1			1						1							1			1	1																												
orbitosphenoid right						1									1				1						1					1				1		1																											
palatine left																				1		1				1							1														1															1	
palatine right																					1		1				1					1																1															1
parasphenoid						1									1			1	1					1	1					1	1																																
parietal left																1		1						1						1						1	1								1										1				1				
parietal right																	1		1						1						1				1			1								1										1			1				
postorbital left																		1		1		1		1											1						1														1								
postorbital right																			1		1		1		1											1						1														1							
prearticular left	1		1				1																																														1				1			1			
prearticular right		1		1				1																																														1				1			1		
prefrontal left																		1				1						1									1																										
prefrontal right																			1				1						1									1																									
premaxilla left																										1		1																1																		1	
premaxilla right																											1		1														1																				1
prootic left					1	1										1								1											1																				1								
prootic right					1	1											1								1											1																				1							
pterygoid left						1					1		1																			1																	1													1	
pterygoid right						1						1		1																			1																	1													1
quadrate left			1													1																															1				1				1					1			
quadrate right				1													1																															1				1				1					1		
quadratojugal left																				1																													1						1								
quadratojugal right																					1																													1						1							
splenial left	1						1		1																														1																		1						
splenial right		1						1		1																														1																		1					
squamosal left																1																			1		1								1				1		1												
squamosal right																	1																			1		1								1				1		1											
supradentary left							1		1																														1														1										
supradentary right								1		1																														1														1									
supraoccipital																1	1																		1	1																											
surangular left	1		1				1		1																														1										1														
surangular right		1		1				1		1																														1										1													
vomer left																										1						1											1				1																1
vomer right																											1						1											1				1														1	

Gallus gallus	angular left	angular right	articular left	articular right	beak	braincase	dentary left	dentary right	jugal left	jugal right	lateral ethmoid left	lateral ethmoid right	palatine left	palatine right	parasphenoid left	parasphenoid right	postfrontal left	postfrontal right	prearticular left	prearticular right	prefrontal left	prefrontal right	pterygoid left	pterygoid right	quadrate left	quadrate right	quadratojugal left	quadratojugal right	splenial left	splenial right	supraangular left	supraangular right	vomer left	vomer right
angular left			1				1												1										1		1			
angular right				1				1												1										1		1		
articular left	1																		1						1						1			
articular right		1																		1						1						1		
beak									1	1			1	1							1	1											1	1
braincase															1	1	1	1			1	1			1	1								
dentary left	1							1																					1		1			
dentary right		1					1																							1		1		
jugal left					1																						1							
jugal right					1																							1						
lateral ethmoid left																					1													
lateral ethmoid right																						1												
palatine left																							1										1	
palatine right																								1										1
parasphenoid left						1																	1											
parasphenoid right						1																		1										
postfrontal left						1																												
postfrontal right						1																												
prearticular left	1		1																										1					
prearticular right		1		1																										1				
prefrontal left					1	1					1																							
prefrontal right					1	1						1																						
pterygoid left													1		1										1								1	
pterygoid right														1		1										1								1
quadrate left			1			1																	1				1							
quadrate right				1		1																		1				1						
quadratojugal left									1																1									
quadratojugal right										1																1								
splenial left	1						1												1															
splenial right		1						1												1														
supraangular left	1		1				1																										
supraangular right		1		1				1																									
vomer left					1								1										1											1
vomer right					1									1										1									1	

Alligator mississippiensis	angular left	angular right	articular left	articular right	basioccipital	basisphenoid	coronoid left	coronoid right	dentary left	dentary right	ectopterygoid left	ectopterygoid right	exoccipital left	exoccipital right	frontal left	frontal right	jugal left	jugal right	lacrimal left	lacrimal right	laterosphenoid left	laterosphenoid righ	maxilla left	maxilla right	nasal left	nasal right	palatine left	palatine right	palpebral left	palpebral right	parietal left	parietal right	postorbital left	postorbital right	prefrontal left	prefrontal right	premaxilla left	premaxilla right	prootic left	prootic right	pterygoid left	pterygoid right	quadrate left	quadrate right	quadratojugal left	quadratojugal right	splenial left	splenial right	squamosal left	squamosal right	supraoccipital	surangular left	surangular right
angular left			1				1		1																																						1					1	
angular right				1				1		1																																					1					1
articular left	1																																										1									1	
articular right		1																																										1									1
basioccipital						1							1	1																																							
basisphenoid					1								1	1							1	1																	1	1	1	1	1	1									
coronoid left	1																																														1					1	
coronoid right		1																																													1					1
dentary left	1																																														1					1	
dentary right		1																																													1					1
ectopterygoid left																	1						1										1								1												
ectopterygoid right																		1						1										1								1											
exoccipital left					1	1								1																						1				1						1		1		
exoccipital right					1	1							1																								1				1						1	1		
frontal left																1					1				1						1		1		1																	
frontal right															1							1				1						1		1		1																
jugal left											1								1				1																						1								
jugal right												1								1				1																					1							
lacrimal left																	1						1												1																		
lacrimal right																		1						1												1																	
laterosphenoid left						1									1																		1						1		1		1										
laterosphenoid right						1										1																	1						1		1		1									
maxilla left											1						1		1					1	1		1										1																
maxilla right												1						1		1			1			1		1										1															
nasal left															1								1			1									1		1																
nasal right																1								1	1											1		1															
palatine left																							1					1							1						1												
palatine right																								1			1									1						1											
palpebral left																																			1																		
palpebral right																																				1																	
parietal left															1																		1						1											1		1		
parietal right																1																		1						1											1	1		
postorbital left											1					1						1										1												1		1				1				
postorbital right												1					1						1										1												1		1				1			
prefrontal left															1					1						1		1		1												1												
prefrontal right																1					1						1		1		1												1											
premaxilla left																							1		1														1															
premaxilla right																								1		1												1																
prootic left						1							1									1										1																				1		
prootic right						1								1									1										1																			1		
pterygoid left						1					1										1						1								1							1	1										
pterygoid right						1						1										1						1								1					1			1									
quadrate left				1			1							1									1												1								1				1				1				
quadrate right					1		1								1									1												1								1				1				1			
quadratojugal left																	1																1										1										
quadratojugal right																		1																1										1									
splenial left	1						1		1																																												1	
splenial right		1						1		1																																											1
squamosal left													1																			1		1										1								1		
squamosal right														1																		1		1										1							1		
supraoccipital													1	1																	1	1								1	1									1	1			
surangular left	1		1				1		1																																						1						
surangular right		1		1				1		1																																					1					

Dermochelys coriacea	angular left	angular right	articular left	articular right	basioccipital	coronoid left	coronoid right	dentary left	dentary right	exoccipital left	exoccipital right	frontal left	frontal right	jugal left	jugal right	maxilla left	maxilla right	opisthotic left	opisthotic right	palatine left	palatine right	parabasisphenoid	parietal left	parietal right	postfrontal left	postfrontal right	prearticular left	prearticular right	prefrontal left	prefrontal right	premaxilla left	premaxilla right	prootic left	prootic right	pterygoid left	pterygoid right	quadrate left	quadrate right	quadratojugal left	quadratojugal right	squamosal left	squamosal right	supra-/surangular left	supra-/surangular righ	supraoccipital	vomer
angular left			1						1																			1																1			
angular right				1						1																			1																1		
articular left	1																										1										1						1			
articular right		1																										1										1						1		
basioccipital										1	1											1														1	1									1	
coronoid left								1																			1																1			
coronoid right									1																			1																1		
dentary left	1					1			1																		1																1			
dentary right		1					1	1																				1																1		
exoccipital left					1													1																											1	
exoccipital right					1														1																										1	
frontal left													1										1		1					1																
frontal right												1												1		1					1															
jugal left																1									1														1		1					
jugal right																	1									1														1		1				
maxilla left														1						1					1				1		1															1
maxilla right															1						1					1				1		1														1
opisthotic left										1																							1				1								1	
opisthotic right											1																							1				1							1	
palatine left																1					1														1											1
palatine right																	1			1																1										1
parabasisphenoid					1																												1	1	1	1										
parietal left												1												1	1																1				1	
parietal right													1										1			1																1			1	
postfrontal left												1		1		1							1						1												1					
postfrontal right													1		1		1							1						1												1				
prearticular left	1		1			1		1																																					
prearticular right		1		1			1		1																																				
prefrontal left												1				1									1					1																
prefrontal right													1				1									1			1																	
premaxilla left																1																1														1
premaxilla right																	1														1															1
prootic left																		1				1													1		1								1	
prootic right																			1			1														1		1							1	
pterygoid left					1															1		1											1			1	1									1
pterygoid right					1																1	1												1	1			1								1
quadrate left			1															1															1		1				1		1					
quadrate right				1															1															1		1				1		1				
quadratojugal left														1																							1				1					
quadratojugal right															1																							1				1				
squamosal left														1									1		1												1		1							
squamosal right															1									1		1												1		1						
supra-/surangular left	1		1			1		1																																					
supra-/surangular right		1		1			1		1																																				
supraoccipital					1					1	1							1	1				1	1									1	1												
vomer																1	1			1	1										1	1			1	1										

Sphenodon punctatus	angular left	angular right	articular left	articular right	basioccipitale	basisphenoid	coronoid left	coronoid right	dentary left	dentary right	ectopterygoid left	ectopterygoid right	epipterygoid left	epipterygoid right	exoccipital left	exoccipital right	frontal left	frontal right	jugal left	jugal right	maxilla left	maxilla right	nasal left	nasal right	opisthotic left	opisthotic right	palatine left	palatine right	parietal left	parietal right	postfrontal left	postfrontal right	postorbital left	postorbital right	prearticular left	prearticular right	prefrontal left	prefrontal right	premaxillary left	premaxillary right	prootic left	prootic right	pterygoid left	pterygoid right	quadrate left	quadrate right	quadratojugal left	quadratojugal right	septomaxilla left	septomaxilla right	squamosal left	squamosal right	supraoccipitale left	supraoccipitale right	surangular left	surangular right	vomer left	vomer right
angular left									1																										1																				1			
angular right										1																										1																				1		
articular left																																			1										1										1			
articular right																																				1										1										1		
basioccipitale						1									1	1									1	1																																
basisphenoid					1								1	1																											1	1	1	1														
coronoid left									1																										1																				1			
coronoid right										1																										1																				1		
dentary left	1						1			1																																																
dentary right		1						1	1																																																	
ectopterygoid left																			1		1						1																1															
ectopterygoid right																				1		1						1																1														
epipterygoid left						1																							1												1		1		1													
epipterygoid right						1																								1												1		1		1												
exoccipital left					1																				1																												1					
exoccipital right					1																					1																												1				
frontal left																		1					1						1		1						1																					
frontal right																	1							1						1		1						1																				
jugal left											1										1						1						1														1				1							
jugal right												1										1						1						1														1				1						
maxilla left											1								1				1				1										1		1										1									
maxilla right												1								1				1				1										1		1										1								
nasal left																	1				1			1													1		1																			
nasal right																		1				1	1															1		1																		
opisthotic left					1										1																										1				1						1							
opisthotic right					1											1																										1				1						1						
palatine left											1								1		1																1						1														1	
palatine right												1								1		1																1						1														1
parietal left													1				1													1	1		1								1										1		1					
parietal right														1				1											1			1		1								1										1		1				
postfrontal left																	1												1				1																									
postfrontal right																		1												1				1																								
postorbital left																			1										1		1																				1							
postorbital right																				1										1		1																				1						
prearticular left	1		1				1																																																1			
prearticular right		1		1				1																																																1		
prefrontal left																	1				1		1				1																															
prefrontal right																		1				1		1				1																														
premaxillary left																					1		1																	1									1								1	
premaxillary right																						1		1															1											1								1
prootic left						1							1												1				1																								1					
prootic right						1								1												1				1																								1				
pterygoid left						1					1		1														1																	1	1						1						1	
pterygoid right						1						1		1														1															1			1						1						1
quadrate left			1										1												1																		1				1				1							
quadrate right				1										1												1																		1				1				1						
quadratojugal left																			1																										1						1							
quadratojugal right																				1																										1						1						
septomaxilla left																					1																		1																		1	
septomaxilla right																						1																		1																		1
squamosal left																			1						1				1				1										1		1		1						1					
squamosal right																				1						1				1				1										1		1		1						1				
supraoccipitale left															1														1												1										1							
supraoccipitale right																1														1												1										1						
surangular left	1		1				1		1																										1																							
surangular right		1		1				1		1																										1																						
vomer left																											1												1				1						1									1
vomer right																												1												1				1						1							1	

Didelphis virginiana	premaxilla left	premaxilla right	nasal left	nasal right	maxilla left	maxilla right	lacrimal left	lacrimal right	ethmoid-vomer	palatine left	palatine right	sphenoid	pterygoid left	pterygoid right	frontal	parietal left	parietal right	zygomatic left	zygomatic right	petromastoid left	petromastoid right	tympanic left	tympanic right	squamosal left	squamosal right	occipital	supraoccipital	mandible left	mandible right	stapes left	stapes right	incus left	incus right	malleus left	malleus right
premaxilla left		1	1		1																														
premaxilla right	1			1		1																													
nasal left	1			1	1										1																				
nasal right		1	1			1									1																				
maxilla left	1		1			1	1		1	1					1			1																	
maxilla right		1		1	1			1	1		1				1				1																
lacrimal left					1				1	1		1			1			1																	
lacrimal right						1			1		1	1			1				1																
ethmoid-vomer					1	1	1	1		1	1	1			1																				
palatine left					1		1		1		1	1	1		1																				
palatine right						1		1	1	1		1		1	1																				
sphenoid							1	1	1	1	1		1	1	1	1	1	1	1	1	1			1	1	1									
pterygoid left										1		1																							
pterygoid right											1	1																							
frontal			1	1	1	1	1	1	1	1	1	1				1	1							1	1										
parietal left												1			1		1							1			1								
parietal right												1			1	1									1		1								
zygomatic left					1		1					1												1											
zygomatic right						1		1				1													1										
petromastoid left												1												1		1									
petromastoid right												1													1	1									
tympanic left																														1					
tympanic right																															1				
squamosal left												1			1	1		1		1						1	1	1							
squamosal right												1			1		1		1		1					1	1		1						
occipital												1								1	1			1	1		1								
supraoccipital																1	1							1	1	1									
mandible left																								1					1						
mandible right																									1			1							
stapes left																						1										1			
stapes right																							1										1		
incus left																														1				1	
incus right																															1				1
malleus left																																1			
malleus right																																	1		

Online Supplement 2. Supplementary Figures

Tyrannosaurus rex

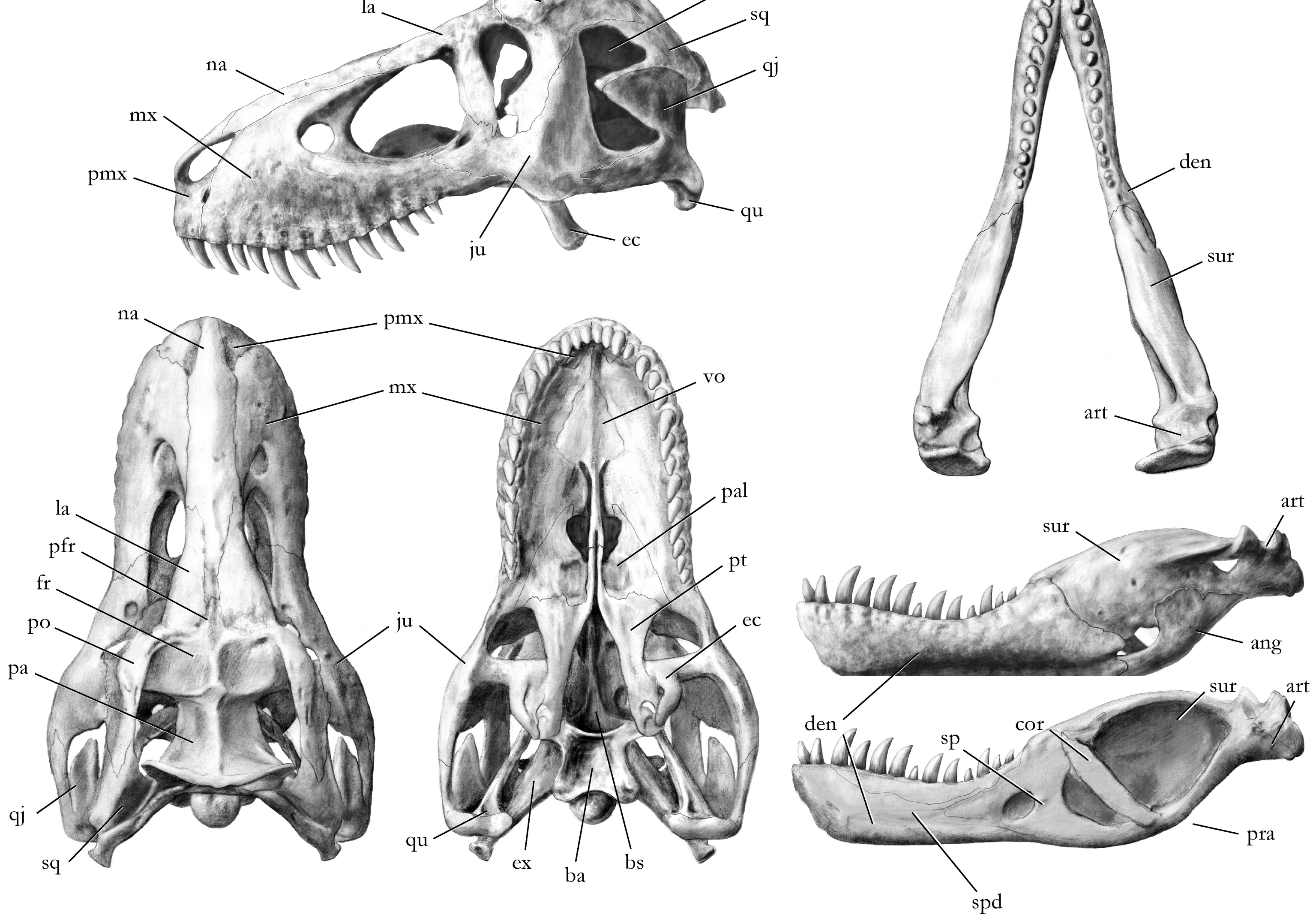

Gallus gallus

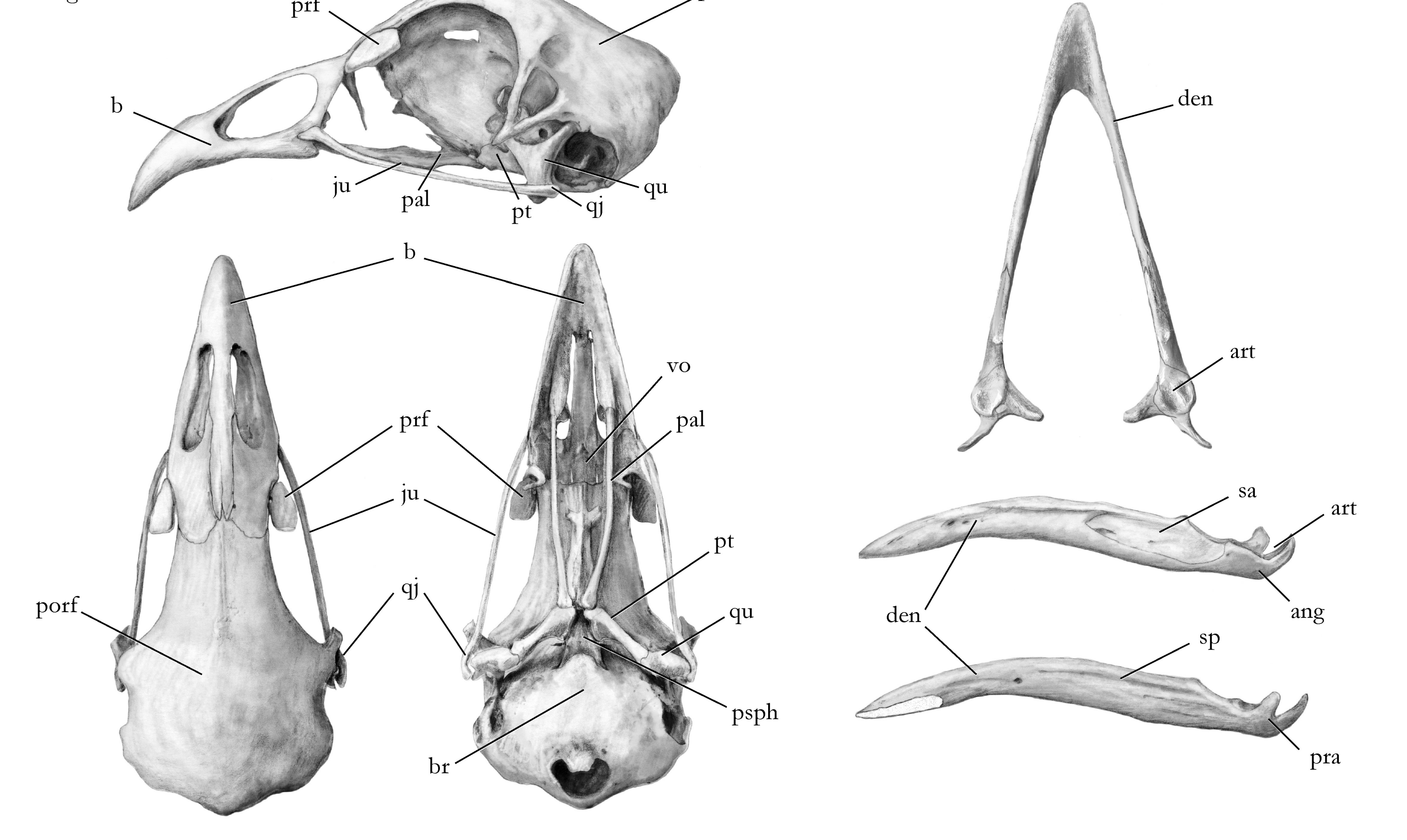

Alligator mississippiensis

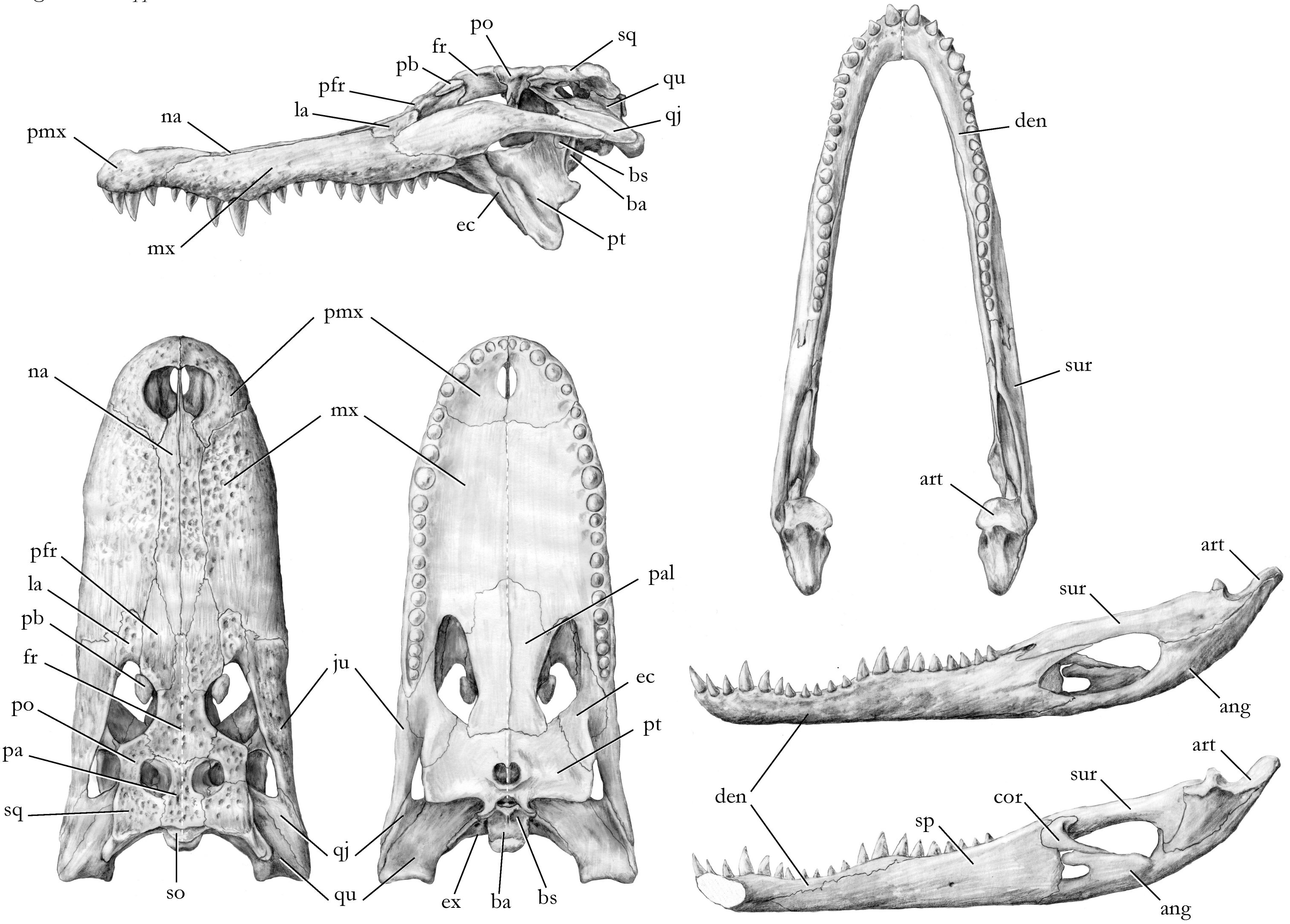

Dermochelys coriacea

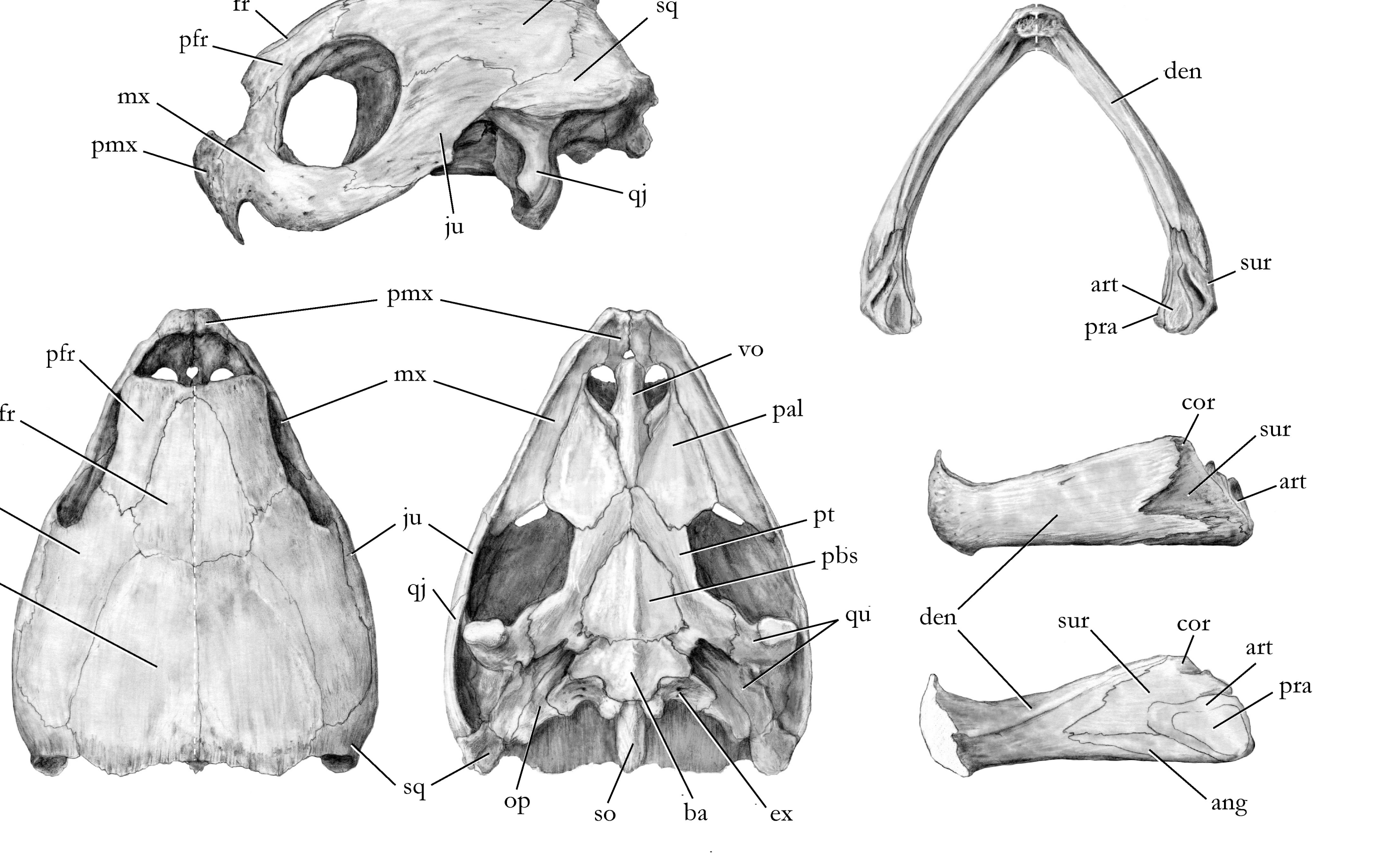

Sphenodon punctatus

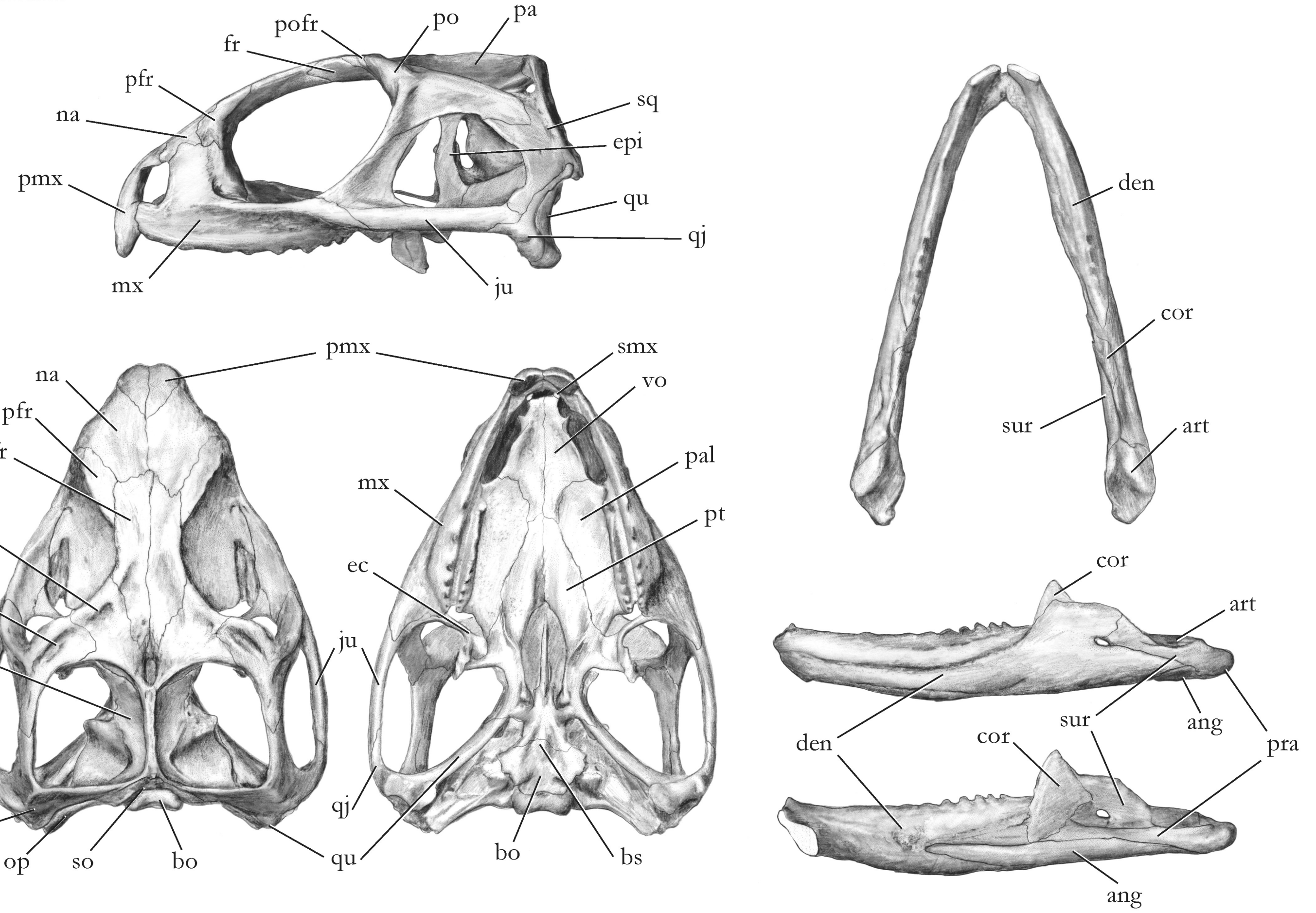

Didelphis virginiana

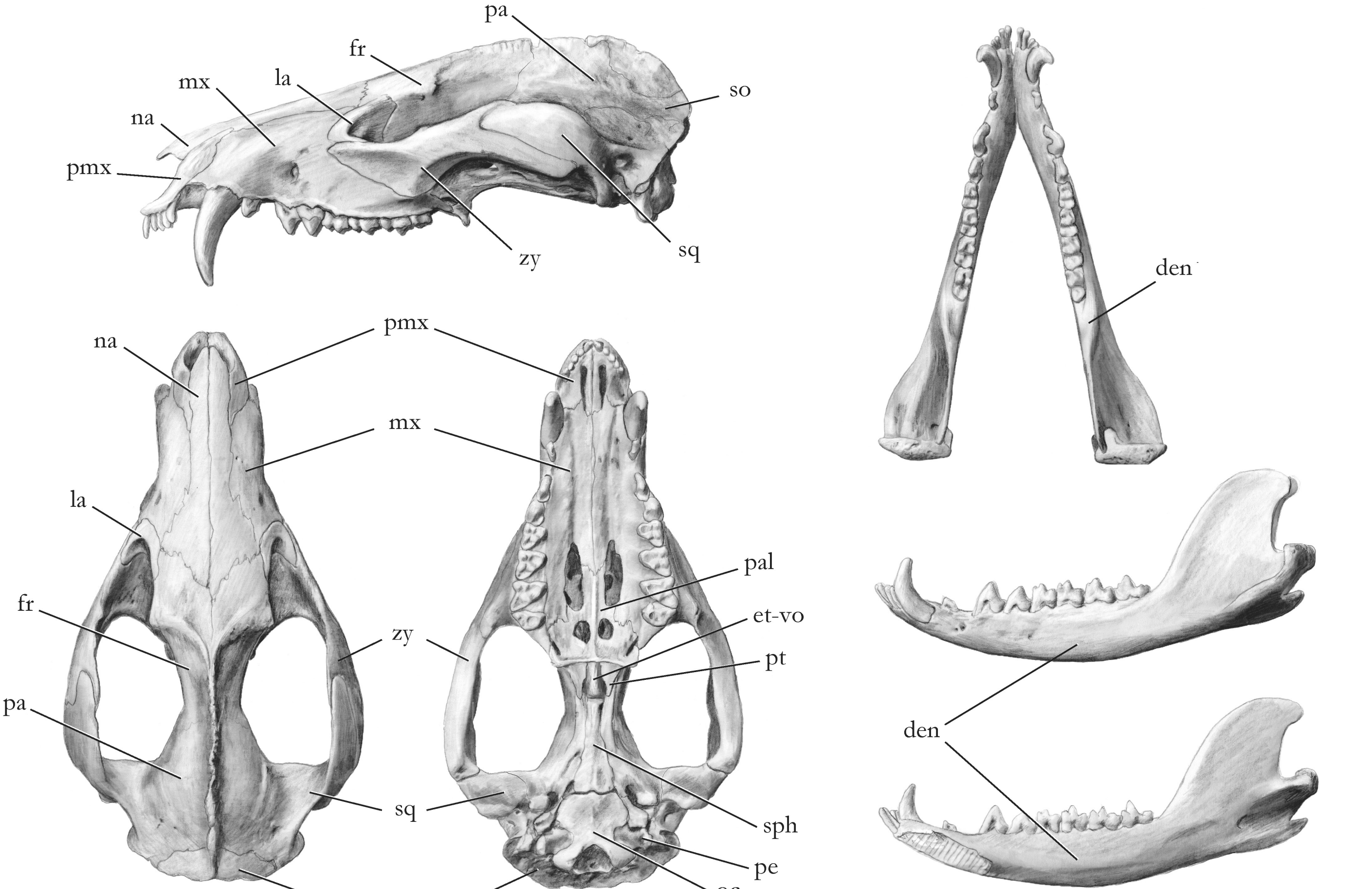

Online Supplement 3. R script

```
#####################################################################################
#
#
# Supplementary Information (R script)
#
# Title: Unique skull network complexity of Tyrannosaurus rex among land vertebrates
# Authors: Ingmar Werneburg et al.
# Date: 2018
#
#####################################################################################
##

# required packages
library(XLConnect)  # load adjacency matrices from Excel to R
library(igraph)     # network analysis
library(ape)        # phylogenetic analysis and hierarchical cluster manipulation
library(phytools)   # phylogenetic analysis and hierarchical cluster manipulation
library(geiger)     # phylogenetic analysis and hierarchical cluster manipulation
library(adephylo)   # phylogenetic analysis and hierarchical cluster manipulation
library(phylobase)  # phylogenetic analysis and hierarchical cluster manipulation
library(picante)    # phylogenetic analysis and hierarchical cluster manipulation

# load adjacency matrices and create networks
species <- c("Tyrannosaurus", "Gallus", "Alligator", "Dermochelys", "Sphenodon", "Didelphis")
mat.list <- mapply(readWorksheetFromFile, "Reptile_Skull_Nets_19Oct2017.xls",
sheet=1:length(species), rownames=1, check.names=FALSE)
adj.list <- lapply(mat.list, data.matrix, rownames.force=TRUE)
pre.graph.list <- lapply(adj.list, graph_from_adjacency_matrix, mode="undirected")
graph.list <- pre.graph.list
names(graph.list) <- species

# generating a file with bone labels
labels2save <- list()
for (i in 1:length(species)){
num_label <- 1:vcount(graph.list[[i]])
names(num_label) <- V(graph.list[[i]])$name
labels2save[[i]] <- paste(num_label, names(num_label), sep=" ")
}
text.out <- c(species[1], labels2save[[1]], species[2], labels2save[[2]], species[3], labels2save[[3]],
        species[4], labels2save[[4]], species[5], labels2save[[5]], species[6], labels2save[[6]])
fileConn<-file("Bone Labels Reptiles.txt")
write(text.out, file=fileConn, ncolumns=1)
close(fileConn)


## Anatomical Network Analysis

# parameters
parameters <- matrix(NA, nrow=length(species), ncol=6) # Table in Figure 1B.
rownames(parameters) <- species
colnames(parameters) <- c("N", "K", "D", "C", "L", "H")
```

```
parameters[,1] <- mapply(vcount, graph.list)
parameters[,2] <- mapply(ecount, graph.list)
parameters[,3] <- mapply(edge_density, graph.list)
parameters[,4] <- mapply(transitivity, graph.list, type="average", isolates="NaN")
parameters[,5] <- mapply(mean_distance, graph.list, directed=FALSE)
heterogeneity = function(graph){
  deg = degree(graph)
  res = sd(deg, na.rm=TRUE)/mean(deg, na.rm=FALSE)
}
parameters[,6] <- mapply(heterogeneity, graph.list)

# mapping parameters in the phylogeny (Figure 1C-H)
s <-
"(((((Tyrannosaurus:200,Gallus:200):45,Alligator:245):18,Dermochelys:263):21,Sphenodon:284):36,D
idelphis:320);"
cat(s, file = "ex.tre", sep = "\n")
tree <- read.tree("ex.tre")
plotname <- c("Number of nodes (N)", "Number of links (K)", "Density of connections (D)",
"Clustering coefficient (C)", "Shortest path length (L)", "Heterogeneity of connections (H)")
for (i in 1:length(plotname)){
  trait <- as.vector(parameters[,i])
  names(trait) <- species
  fancyTree(tree, type = "phenogram95", x = trait, spread.cost = c(1,0), spread.labels=FALSE,
        xlab = "Time from origin (Myr)", ylab = plotname[i])
  phenogram(tree, x = trait, spread.cost = c(1,0), add = TRUE, ftype = "off")
}


## Community structure (modularity)

# function: GTOM (from http://labs.genetics.ucla.edu/horvath/GTOM/old/gtom.R)
GTOMmdist1 = function(adjmat1,m=1){
  if (m!=round(abs(m))){
    stop("m must be a positive integer", call.=TRUE);}
  if (any(adjmat1!=0 & adjmat1!=1)){
    stop("The adjacency matrix must be binary", call.=TRUE);}
  B <- adjmat1;
  if (m>=2) {
    for (i in 2:m) {
      diag(B) <- diag(B) + 1;
      B = B %*% adjmat1;}}   # number of paths with length at most m connecting each pair
  B <- (B>0);                   # m-step reachability matrix
  diag(B) <- 0;                 # exclude each node being its own neighbor
  B <- B %*% B;                 # number of common k-step neighbors that each pair of nodes share
  Nk <- diag(B);                # number of common k-step neighbors that each node possesses
  B <- B +adjmat1;
  diag(B) <- 1;
  denomTOM=outer(Nk,Nk,FUN="pmin")+1-adjmat1;
  diag(denomTOM) <- 1;
  1 - B/denomTOM                # turn the GTOM matrix into a dissimilarity
}
```

```
# function: prepare adjacency matrices
na.zero <- function (x) {
  x[is.na(x)] <- 0
  return(x)
}

# calculate GTOM and hierarchical clustering
adj.list <- lapply(adj.list, na.zero)
tom.list <- lapply(adj.list, GTOMmdist1, m=1)
dist.tom.list <- lapply(tom.list, as.dist)
hclust.list <- lapply(dist.tom.list, hclust, method="average")
for (i in 1:length(species)){hclust.list[[i]]$labels <- V(graph.list[[i]])$name}
phylo.list <- lapply(hclust.list, as.phylo)

# function: jackknife of modularity Q value
jackknife_Q = function(graph, membership){
  Qi <- vector()
  for (j in 1:ecount(graph)){
    g <- delete_edges(graph, j)
    Qi[j] <- modularity(g, membership)
  }
  ss <- sum((Qi-mean(Qi))^2)
  n <- (ecount(graph)-1)/ecount(graph)
  Q.error <- sqrt(n*ss)
  return(Q.error)
}

# best partition based on Q max
best.partition <- vector()
for (i in 1:length(species)){
  Qvalue <- vector()
  for (j in 1:vcount(graph.list[[i]])){
    Qvalue[j] <- modularity(graph.list[[i]], cutree(hclust.list[[i]], k=j))
  }
  best.partition[i] <- which(Qvalue==max(Qvalue))
}

# function: Wilcox test to evaluate modules
community.significance.test <- function(graph, vids, ...) {
  subgraph <- induced_subgraph(graph, vids)
  indegrees <- degree(subgraph)
  outdegrees <- degree(graph, vids) - indegrees
  wilcox.test(indegrees, outdegrees, alternative="greater")
}

# partition based on statistical significance
Ho_modules_pvalue <- list()
for (i in 1:length(species)){
  graph <- graph.list[[i]]
  tree <- phylo.list[[i]]
```

```
  tipN <- vcount(graph)
  intN <- tipN-1
  Ho_modules_pvalue_v <- vector()
  for (j in 1:intN){
    Ho_m <- extract.clade(tree, node=(tipN+j)) # get all subtrees
    is_tip <- Ho_m$edge[,2] <= length(Ho_m$tip.label)
    ordered_tips <- Ho_m$edge[is_tip, 2]
    who <- Ho_m$tip.label[ordered_tips]
    index <- which((V(graph)$name %in% who)==TRUE)
    Ho_mp <- community.significance.test(graph, vids=index)
    Ho_modules_pvalue_v[j] <- Ho_mp$p.value
  }
  Ho_modules_pvalue[[i]] <- Ho_modules_pvalue_v
}

# dendrogram plots (Figures 2 and 3)
for (i in 1:length(species)){
  plotTree(phylo.list[[i]], lwd=1, mar=c(4.1,1.1,1.1,1.1))
  # p-value groups
  sign <- which(Ho_modules_pvalue[[i]]<0.05)
  bs <- Ho_modules_pvalue[[i]][sign]
  co <- c("black", "grey", "white")
  p <- character(length(sign))
  p[bs < 0.001] <- co[1]
  p[bs >= 0.001 & bs < 0.01] <- co[2]
  p[bs >= 0.01 & bs < 0.05] <- co[3]
  nodelabels(node=(sign+vcount(graph.list[[i]])), pch=21, bg=p, cex=2)
  nodelabels(phylo.list[[i]]$node.label, node=(sign+vcount(graph.list[[i]])), adj=c(1.1,-
0.7),frame="none")
  # Qmax groups
  obj <- ltt(phylo.list[[i]], plot=FALSE)
  k <- best.partition[i]
  h <- mean(obj$times[c(which(obj$ltt==k), which(obj$ltt==(k+1)))])
  lines(rep(h,2), par()$usr[3:4], col="red", lty="dashed")
  title(main=paste("Modularity of the skull network of", species[i]))
}

# groping bones by module
members.list <- list()
for (i in 1:length(species)){
  n <- 2
  p <- 0
  end <- FALSE
  while (end==FALSE){
    members <- cutree(hclust.list[[i]], n)
    for (j in 1:n){p[j] <- community.significance.test(graph.list[[i]], vids=(members==j))$p.value}
    if (all(p<0.05)==TRUE){
      n <- n+1
    } else {
      n <- n-1
      p <- 0
```

```
      members <- cutree(hclust.list[[i]], n)
      for (j in 1:n){p[j] <- community.significance.test(graph.list[[i]], vids=(members==j))$p.value}
      end <- TRUE
    }
  }
  members.list[[i]] <- members
}

# modules information (Figure 1B)
modules <- matrix(NA, nrow=length(species), ncol=4)
rownames(modules) <- species
colnames(modules) <- c("S Modules", "Q Modules", "Qmax", "Qmax error")
for (i in 1:length(species)){
  modules[i,1] <- max(members.list[[i]])
  membership <- cutree(hclust.list[[i]], k=best.partition[i])
  modules[i,2] <- best.partition[i]
  modules[i,3] <- modularity(graph.list[[i]], membership)
  modules[i,4] <- jackknife_Q(graph.list[[i]], membership)
}
```

Online Supplement 4. ex.tre (phylogenetic tree file)

```
(((((Tyrannosaurus:200,Gallus:200):45,Alligator:245):18,Dermochelys:263):
21,Sphenodon:284):36,Didelphis:320);
```
